# Practical Medicine

**Published:** 1879-01

**Authors:** 


### Summary.

*Collaborators:*

DR. H. GRADLE, DR. L. W. CASE, DR. R. PARK,
DR. R. TILLEY, DR. D. R. BROWER.

### PRACTICAL MEDICINE.

TREATMENT OF GENERAL PROGRESSIVE PARALYSIS (DEMENTIA PARALYTICA). *Medical & Surgical Reporter.* Translated from the German of Prof. Ludwig Meyer by Dr. Liebmann, Nov. 2, 1878.

Prof. Meyer details a method of treatment that has been followed by remarkable success in this hitherto regarded incurable disease. He regards the disease as a chronic encephalitis, limited to the cortical layer of the convexity of the anterior half of the great hemispheres. He combats this condition by vigorous counter-irritation established as near as possible to the seat of the affection. He lays great stress upon his method of irritation, and as this treatment should be instituted in the very beginning, that is, before the necessity has arrived for the confinement in an insane hospital, we give it in full.

"The hair should be shaved off from the scalp in the region of the great fontanelle, to the extent of half a hand, and then as much tartar emetic ointment (1:4 Germ. pharm.) be rubbed in the central portion of the shaved surface as would cover a silver dollar. Much depends on the first application being accurate and energetic. A portion of the salve, as big as a pea, I caused to be rubbed on twice, by means of a firm and smooth pledget of linen of the size of a finger, and then I covered the reddened surface with a piece of linen saturated with the ointment. The second application, which has to take place twenty-six hours later, ought to be made with the greatest care and precaution, in order to save the already loosened epidermis. Such denudation would cause considerable pain, and besides, would not benefit the procedure any, for this latter's success depends entirely on the feasibility of bringing the scalp, in its greatest possible depth, to swell and gradually to slough. Two applications, as a rule, suffice, but, to be cautious, it is well to spread, on the third day, a small portion of the ointment on the already elevated surface. During the third and fourth day the swelling increases, extends to the frontal region, and even to the face—rarely to the occiput and neck. As soon as the swelling increases, warm poultices should be applied freely. In a few days the integuments are detached by the abundant suppuration. The whole procedure requires on an average fourteen days, and leaves a regular, round, deep and copiously suppurating sore, which, by means of the basilic ointment, should be kept open for two or three months. During this whole time, and beyond it, if tolerated well, the patients are put upon iodide of potassium, in moderate doses (3–5, 0, aq. dest. 180,0, a tablespoonful four times a day). The diet, especially during the first weeks of this treatment, ought to be easily digestible, but vigorous. The patient should be sent into the open air as soon and as often as possible; should, if his strength allows it, do light work in the garden, but should be guarded against the heat of the summer's sun. Baths in any form are to be avoided, for they are, under these conditions, only too apt to produce congestion of the head."

Prof. M. has applied this treatment to seventeen cases of this disease. Of this number, two only remained under treatment fourteen days, one of these manifesting remarkable improvement; of the remaining fifteen, eight were cured; of these eight, one experienced a relapse from the too early abandonment of the treatment. A detailed history is given of the eight successful cases.

The article closes with a warning against a too energetic anti-syphilitic treatment.

